# Motherhood and medicinal cannabis

**DOI:** 10.1111/dar.14027

**Published:** 2025-02-18

**Authors:** Vinuli Withanarachchie, Marta Rychert, Chris Wilkins

**Affiliations:** ^1^ Shore & Whāriki Research Centre, College of Health Massey University Auckland New Zealand

**Keywords:** maternal health, medical marijuana, medicinal cannabis, stigma, women

## Abstract

**Introduction:**

Women are emerging as a key demographic for medicinal cannabis (MC) use in countries that have implemented MC reforms. However, research on mothers' experiences of consuming MC remains limited beyond studies on perinatal outcomes. This study explores mothers' diverse experiences of consuming MC in New Zealand under the legal MC scheme.

**Methods:**

Interviews with 15 mothers using MC via prescriptions, the illegal market or both in the last 12 months. Thematic analysis focused on MC use in parenting, MC conversations with children, societal stigma and risks.

**Results:**

Mothers reported MC as an important facilitator of their ability to positively parent their children, enabling them to manage their own health needs (i.e., anxiety, endometriosis and arthritis). High costs of legal products hindered access to MC. Participants expressed unique risks that mothers face accessing the unregulated market for MC like being deemed a ‘bad mother’ and losing custody of children. Stigma was countered with narratives of empowerment through proactive MC conversations with children and agency by self‐medicating with MC despite the judgement they may face for being a parent that uses cannabis.

**Discussion and Conclusions:**

Mothers felt managing their health with MC allowed them to be more present parents and better tolerate the stressors of motherhood. In‐depth exploration of discussing MC with children and anticipating these conversations was a novel finding. Most mothers tried to destigmatise MC in conversations by classifying it in the same category as other medications and discussing its therapeutic benefits. Few were cautious about having these conversations too early.

## 1 INTRODUCTION

Jurisdictions in a growing number of countries have implemented medicinal cannabis (MC) schemes to provide legal pathways for patients to access quality‐controlled products, including in Australia, Canada, the Netherlands, certain states in the United States, and New Zealand (NZ) [1]. NZ's Medicinal Cannabis Scheme (NZMCS) came into effect 1 April 2020, with provision for pharmaceutical‐dosage and herbal cannabis on prescription [2]. The current scientific clinical trial evidence base for MC is growing, however, there are currently only a few indications for which there is clear evidence of efficacy [1]. There is moderate evidence that cannabinoids may reduce symptoms of multiple sclerosis, reduce chemotherapy‐induced nausea and vomiting, and reduce chronic pain [3, 4]. Cannabidiol (CBD—the non‐intoxicating compound in cannabis) has also proved useful in reducing seizures in two childhood epilepsy syndromes, that is, Dravet and Lennox–Gastaut syndrome [5]. In addition, real‐world evidence studies of MC users report improvements in neuropathic pain, chronic pain, cancer‐related pain, sleep and anxiety [6, 7, 8].

Both in NZ and other countries, women have emerged as a key demographic for MC use, with anecdotal evidence suggesting they are an important customer base for licenced MC clinics [9, 10, 11]. Research at the intersection of women and cannabis has predominantly focused on cannabis use during pregnancy and breastfeeding, often linking consumption to adverse perinatal outcomes, substance abuse and mental health disorders [12, 13, 14]. Two US studies of pregnant and lactating mothers using cannabis found most participants were hesitant to discuss cannabis use with health professionals due to fear of judgement and legal implications [15, 16]. Those who disclosed use were either advised to discontinue or they received mixed health messages, rather than health providers engaging in a more open discussion. In two studies cannabis use while pregnant was associated with negative effects on the child, such as poor neurodevelopment, low birth weight and risks of early cannabis use in youth [17, 18].

Women's use of cannabis has also been reported in terms of therapeutic benefits in a handful of studies. Women self‐report consuming cannabis for pain management, fostering better parent–child relationships and improving their tolerance of parental stress [19, 20, 21]. A study of 40 mothers in the United States found regular use of cannabis for recreational and medicinal purposes helped these women regulate their moods, stay present with their children, and improved their ability to parent [22]. In another US survey of 107 mothers who used cannabis, among the 60% who consumed cannabis regularly, 89% felt it improved their parenting [23]. Furthermore, mothers online have asserted personal counternarratives to cultural norms by advocating for the benefits of cannabis use in motherhood [24, 25, 26]. Finally, mothers procuring MC for their children, often in cases of rare and terminal illnesses such as paediatric epilepsy [27, 28], have been topical in the recent media, contributing to MC reforms through their advocacy [29, 30].

The multifaceted relationship between motherhood and MC is underexplored in the literature, despite women growing as a consumer base for legal MC worldwide. Scholars [31, 32] argue that women who use illegal drugs, noting MC was recently considered an illegal drug, face greater social repercussions than men due to societal expectations of femininity, motherhood and gendered behaviours. Societal pressures and discrimination are heightened for Black, Brown and Indigenous women, and mothers with low‐income or mental health conditions, and cannabis use may form another social identity by which they are further stigmatised, possibly hindering their access to treatment [21]. Koza et al. [33] advocate for research into motherhood and cannabis use from the perspective of parents, affording priority to intersecting axes of identity, that is, gender, race and class, particularly for marginalised women. They argue the need to broaden current narrow constructions of motherhood and cannabis use, to shape future cannabis policy.

This research paper aims to explore mothers' diverse lived experiences with MC in the new legal MC scheme in Aotearoa NZ and contribute to an emerging global debate. This study is part of a larger project exploring women's relationship with MC in NZ.

## 2 METHODS

### 2.1 Participants and recruitment

Women were eligible to participate if they identified as female, were aged over 16 years and had used cannabis for medical reasons legally or illegally in the last 12 months in Aotearoa NZ. In total, 38 participants were recruited for the larger project about women's relationship with MC, of which 15 identified as mothers and were included in this study. Participants provided written consent after the initial phone call and verbal consent at the start of interviews. Interviews were conducted between January and March 2024. The study was promoted via a flyer posted on social media forums, that is, Reddit and Facebook, through the social media profiles of local MC activists, and circulated by women's health centres and groups (i.e., Auckland Women's Centre, Endo Warriors). The recruitment flyer stated that interviews could be conducted face‐to‐face or virtually and would focus on MC experiences, needs and views of the new NZMCS. Women did not need an official medical diagnosis to be eligible to participate. Interested participants were called by the first author to discuss their eligibility. The face‐to‐face interview was conducted at a public cafe, and the virtual interviews via Zoom®. Participants were asked to join with the username ‘Participant’, and not to mention any names or places during the interview. This was an important measure to ensure participants felt comfortable disclosing potential illegal activity (i.e., accessing cannabis outside the prescription scheme) and personal or difficult experiences. This study was approved by the Massey University Human Ethics Ohu Matatika 1 on 29 January 2024 (Application OM1 23/50).

### 2.2 Data collection

Qualitative semi‐structured interviews were chosen as the best approach to understand the diverse lived experiences and personal narratives of the participants in their socially situated worlds [34]. Feminist scholars have contributed to this method by advocating for co‐constructed knowledge through storytelling and meaning making in interviews, moving away from dated notions of the objective researcher [35]. One interview for the current study was conducted face‐to‐face and the remaining 14 were virtual, with the duration of interviews ranging from 55 to 90 min. The 25‐item interview schedule included questions about using MC for health and wellbeing, affordability and access to products, stigma and societal perceptions, use of recreational cannabis and other medicines, and future needs for policy and reform. The interview schedule was informed by a comprehensive literature review and discussions with healthcare providers who had experience with women's MC use, such as naturopaths, herbalists and gynaecologists. The first author conducted, audio‐recorded and transcribed the interviews ad verbatim.

### 2.3 Data analysis

The data was interpreted using the qualitative description approach, then thematically analysed. Initially, the first author used NVivo software to organise and categorise data within the qualitative description approach [36, 37, 38], aiming for a low‐inference interpretation of how mothers used MC to manage their emotional and physical health such as their reasons for MC use and the impacts on their daily functioning. Next, deductive thematic analysis [39] was conducted, and the transcripts were coded according to the interview topics: societal stigma, impacts on parenting, affordability of products and risks. This was followed by an inductive approach [40] to identify additional themes as analysis progressed such as maternal identity, balancing self‐care with caregiving, and narratives of empowerment. The themes were discussed between the first and second authors to sense‐check ideas and consider their multiple interpretations of the data while acknowledging their own positionalities. The second and third authors provided numerous rounds of written feedback on paper drafts. To de‐identify the data, individual participant quotes are represented by P1, P2, P3, etc.

## 3 RESULTS

A total of 15 participants who had children between the ages of 4 and 18 years were included in this study. All were 30 years or older. Eight mothers used cannabis from the illegal unregulated market, five were transitioning from the illegal market to prescription use or using a mix of both, and two mothers had only used prescription MC in the last 12 months. MC usage ranged from multiple times a day to a minimum of 3 days a week. Participant demographics and medical reasons for use are presented in Table 1. All participants used MC to treat co‐morbidities and three identified with multiple ethnicities.

**TABLE 1:** Demographics of participants.

Characteristic	*N*	%
Location		
Auckland (New Zealand's biggest city)	4	26.6
North Island (not Auckland)	7	46.6
South Island	4	26.6
Age		
30–40 years	4	26.6
40–50 years	9	60
50–60 years	2	13.3
Ethnicity		
New Zealand European	10	66.6
Māori	2	13.3
Māori/ New Zealand European	1	6.7
Māori/Scottish	1	6.7
Asian/New Zealand European	1	6.7
Education		
Didn't finish high school	3	20
Certificate/Diploma/Technical Study	7	46.6
Bachelor's Degree	3	20
Master's Degree	2	13.3
Employment		
Full time employed	2	13.3
Part time employed	2	13.3
Self‐employed	4	26.6
Not currently employed	7	46.6
Medical reason		
Gynaecological health—Endometriosis, dysmenorrhea, menopause	6	40
Mental health conditions—Depression, anxiety, premenstrual dysmorphic disorder, bipolar disorder, post‐traumatic stress disorder	6	40
Sleep disorder	5	33.3
Migraines	3	20
Attention‐deficit/hyperactivity disorder	2	13.3
Arthritis	2	13.3
Auto immune disease	1	6.6
Neurological disease	1	6.6
Routes of administration		
Smoke via water pipe or joint	7	46.6
Edibles	6	40
Oils	4	26.6
Vaporiser	3	20
Tea	1	6.7
Topical	1	6.7
Source of supply		
Illegal, unregulated market	8	53.3
Both illegal and prescription	5	33.3
Prescription only	2	13.3

Results are presented via the following themes: using MC as a parenting tool, conversations with children about MC, the risks mothers face as MC consumers, and their experiences and perceptions of societal stigma for using cannabis as mothers.

### 3.1 Parenting tool

This category captured MC consumption as an important tool for participants to manage the unique stressors of motherhood. Mothers consumed MC to relieve their physical health symptoms such as spasms, aching, and cramps. Without the distraction of pain, they believed they could be more present for their children and attend to their needs. Similarly, mothers with mental health and mood conditions such as anxiety, depression, post‐traumatic stress‐disorder, and pre‐menstrual dysphoric disorder felt MC made them calmer, more relaxed and less overwhelmed, which in turn aided their ability to communicate and better connect with their children. A few respondents said consuming MC improved their overall functioning and ability to meaningfully engage in their lives. As a result, they expressed that their kids received better parenting, that is, more ‘happy, funny’ (P10) and ‘empathetic’ (P9), rather than being ‘grumpy’ (P6) prone to ‘snap’ at them (P8).

‘Using cannabis helps me communicate better with my kids. It allows me to manage my emotions and not get so worked up over little things, which in turn opens up conversations with them that might not have happened otherwise’. (Participant 13)

‘I mean, it affects it [parenting] positively and it increases my general functioning. If I'm not in pain, and I'm well‐rested, I can be the type of parent that I aspire to be, which is patient, empathetic, fair, firm, all of those things’. (Participant 9)

Despite strong advocacy for MC use while parenting, all participants emphasised responsible consumption, framing this as reserving MC use for the evening or night time after completing their parental duties or when their children had gone to bed. A couple of participants experienced an internal conflict between the health benefits they received from MC use and the less desirable effects it might have on them that could affect their family life. One mother felt consuming MC made her less attentive to her surroundings. Further, she did not want to expose her children to the idea of smoking cannabis, preferring to use a vaporiser, which she perceived to be healthier. At times, this meant foregoing MC use for long periods as she felt this was the right decision as a parent.

‘I'd leave it till the evening so that I didn't have any tasks to perform, or I didn't have to worry about being present for the kids. It made me think “am I robbing my kids of quality time with me?”’ (Participant 2)

At the intersection of motherhood and low income, singleness and disability exacerbated issues with affordability of legal MC. All mothers in this category had to budget to afford MC products, often purchasing from the illegal, unregulated market or growing it themselves to save money. Their perceptions that legal MC was unaffordable, which affected their perceived ability to care for themselves, meant they felt it was difficult to extend this care to their children and effectively manage the stressors of motherhood. The two common scenarios described by participants were: (i) having to sacrifice or forego their MC to prioritise their children's needs; and (ii) using less and rationing the amount they consumed over a longer period. These participants felt it was unfair that the high cost of legal MC products in Aotearoa NZ meant they had to resort to the illegal market or home grow to afford their medicines, breaking the law in the process.

‘It's the last thing on the budget because as a mum you're always the last thing on the budget. The things that children need come first. Obviously, it's [illegal cannabis] not as expensive as the prescription, but it's still quite expensive. So back then I wouldn't get as much of it as I would really need because the money's not there’. (Participant 14)

‘I'm a single mum, the roof over my head and keeping the lights on and being able to provide for my baby is more important than my medicine. Even though it does help me function, I can still breathe without it, it's just not very comfortable. Yeah, like I've only been able to afford it once in the past eight months’. (Participant 3)

### 3.2 Conversations with children

This category illustrated the empowering conversations participants had with their children about MC, and their plans to have these discussions in the future. Participants promoted the normalisation of MC use to their children in three ways. First, mothers classified cannabis as a medicine in the same category as their other medications. Mothers who consumed prescribed MC felt procuring it from a pharmacy alongside their other medicines normalised and legitimised cannabis as just another medicine to their children. Second, when asked about whether they had discussed MC use with their children, many respondents felt it was important to pre‐emptively inculcate positive messaging about the therapeutic properties of cannabis to their children, how it benefitted them, and explain that though society may view cannabis users in a negative way, their family values supported the use of cannabis for medicinal purposes. All mothers felt hiding their MC consumption would contribute to the stigma and leave room for their children to make the wrong assumptions, or miscommunicate about it to others.

‘I've made sure to educate them about it thoroughly, so they're able to handle anything if someone says something. They're pretty aware about cannabis and its effects, which I think is great. They understand how it helps me, especially when I'm feeling tense …’ (Participant 13)

‘As long as you explain to the children that it's a medicine then it's no different than any other medicine and they don't view it any differently either. They just see it as medicine’. (Participant 14)

Thirdly, some mothers framed MC as a natural product that has healing properties. For NZ European participants, this framing was associated with a holistic way of living, being connected to nature, and understanding how any kind of consumption affects the body. For one Māori mother, MC was understood as an integral part of Rongoa (Māori medicine), therefore she planned to discuss its healing properties with her very young children in the future through a cultural lens. However, she stated that she was comfortable providing them with MC now if they needed it.

‘We're pretty liberal as in we see it as Rongoa and it is a plant and in Maoridom, we use our Rakau for the benefit of our bodies. If they needed access to it for their health, I would absolutely assist with that. Illegally or legally, I don't see it as an issue’. (Participant 5)

Participants with young children were enthusiastic about the prospect of discussing MC with them in the future. Some mothers looked forward to sharing their personal experiences and journeys to de‐stigmatise cannabis, legitimise the use of MC, and guide their children to safely experiment themselves when they were ready. Other mothers were apprehensive about needing to have this conversation as they felt the information may overwhelm their children, or that it was not necessary to disclose more than MC is a medicine. A few participants expressed a preference for their children to ask them about MC when they were ready or discuss it, and when they had reached an age where they were less likely to accidently disclose that information to others.

‘We'll just be very open with her that there is stigma and that comes from historical reasons and from societal values, but our family values don't align with those societal values … and this is the reasons why we've made the choices that we have and we're comfortable with them’. (Participant 9)

‘I think sometimes you verbally vomit on children and end up telling them a lot more than they actually wanted to know. So, I think we would just have to take it at face value, at that time … and just answer the best that we can’. (Participant 12)

### 3.3 Risks

This category captured how mothers who used MC faced different degrees of risk based on their social identities. Several NZ European mothers felt their white race was a privilege that could protect them from harsh treatment or criminality if caught by the police with non‐prescription MC. They also linked their other social identities such as being in employment, married, educated and a functioning member of society as privileges that to some degree protected them from societal judgement as they did not conform to the stereotype of an illegal drug user. This contrasted with rhetoric from one Māori mother who felt the high costs of prescribed MC prevented Māori from accessing products legally, which meant they were forced into illegal access routes. As a result, she believed Māori faced greater risks of prosecution and damage to their personal and professional lives from using MC.

‘White privilege comes in here in that I do think it would be unlikely I would get jail time. If they found it in my possession, there's a little bit of the idea that because of my ethnicity, and my educated vocabulary, I think, conviction would be unlikely’. (Participant 8)

‘The white supremacist system will amplify anything that reinforces the idea of Māori being seen as bad, and this is just another example. It's like another hurdle, pushing us into situations like imprisonment, having our children taken away, and struggling to find work. It's like the perfect chance to blacklist us’. (Participant 5)

Participants also voiced concerns that their identity as single mothers using MC put them at risk of having their children legally removed from their care. Three mothers who were either separated from their children's fathers or in custody negotiations feared that their MC use would be weaponised against them and used as a reason to suggest they were irresponsible parents. This had served as motivation for two of the mothers to transition from the unregulated market to a legal prescription in the last 12 months.

### 3.4 Societal stigma

This final category captured participants' awareness of the societal stigma and negative perceptions attached to cannabis use, irrespective of medicinal purposes. Some participants internalised this stigma due to the previous illegal status of cannabis in NZ and historical portrayals of deviant cannabis use during their upbringing. These mothers questioned whether their use was justifiable, particularly when using MC to relax, improve appetite and sleep better, traits that overlap with the behaviours of recreational cannabis users. Mothers who were using an MC prescription or transitioning to one were less impacted as they felt they were no longer breaking the law and the prescription legitimised cannabis as a medicine for them. Respondents also drew comparisons between using cannabis and alcohol while parenting, describing the adverse impacts the latter has on children and the unfair reputation the former has gained due to dated, societal narratives.

‘Going prescription immediately reduced the amount of stress that I felt around using it as a medicine. I'm a mother, I have always felt that I was doing something bad or doing risky behaviour in terms of being a mother and having three young children … I always found that I was risking that’. (Participant 1)

Respondents felt that cannabis stigma was compounded by the double standard society imposes on mothers. They highlighted the higher expectations and judgement women face compared to men in general, including that cannabis and alcohol use are more widely accepted as a coping mechanism for men. Furthermore, participants articulated how easily mothers can garner a poor reputation in society. As a marginalised group, single mothers and those on low incomes felt they were going to be judged by others, irrespective of their choices, and therefore they reconciled their MC use with their ability to parent effectively.

‘I just smoke a little bit of cannabis. But I know that is definitely looked down upon because as mothers, we can't do anything right. You're a bad mother if you stay at home with your kids and don't go to work and further your career. You're a bad mother if you go to work and leave your kids with daycare … There's no winning’. (Participant 3)

Fear of being judged, having their ability to parent questioned, or experiencing harsher consequences from other parents, teachers and extended family prevented some mothers from discussing their MC consumption with others. In a few instances where participants had openly disclosed their MC use to other mothers, they received positive or interested responses. A couple of mothers stated they would not voluntarily discuss their MC use in general, however, were open to sharing their experiences if approached by other mothers needing MC for themselves or their children.

## 4 DISCUSSION

The aim of this research was to explore mothers' experiences of MC use during the first years of a new MC scheme in NZ [2, 41]. Participants in our study felt that MC is a useful tool to manage the stressors of motherhood and facilitate positive relationships with their children [19, 22, 27]. They reported using MC to manage their physical health symptoms, regulate their moods [42, 43] and improve their general wellbeing [44, 45], in turn leading to better communication and connection with their children, as well as improved capacity to tolerate misbehaviour and general fatigue. Participants' interest in MC use reflects a general trend in MC uptake by women to treat a range of general and gynaecological conditions [45, 46, 47]. Furthermore, participants felt that being able to manage their physical pain and mental distress with MC meant they were in a better mood and more present. In turn, they were better able to manage the stressors of motherhood, and their children received more positive interactions with them. While cannabis use may make some individuals feel creative, inspired, good, relaxed and inspired—potentially aiding positive parenting as illustrated in our study—many consumers may still feel that it is unsuitable to use around children [48].

Overall, the majority of recreational cannabis users experience low health risks even with long‐term exposure [49]. Individuals who consume cannabis infrequently may experience positive acute effects from the tetrahydrocannabinol (THC) component such as feelings of euphoria and destress. Occasional use may also cause temporary cognitive impairment affecting motoric coordination increasing the risk of vehicle accidents [50]. The risks of health harms from cannabis use increase with certain factors such as more frequent use (e.g., daily), early initiation (e.g., before age 15) and consuming higher THC potent products. Scholars have found that long‐term or frequent use (daily or near daily use) of cannabis is a risk factor for cannabis use disorder, depression and other mental health conditions. Yet, pre‐existing genetic susceptibility is also an important factor in cannabis related mental health issues [49, 51].

For MC consumers, medical assessment for suitability for MC and supervision of use may reduce the risks of unwanted effects. However, it is important to consider that the efficacy of cannabis treatment varies by condition and the impacts of long‐term MC use (daily or near daily use) are still emerging. In NZ, a survey of 3634 MC consumers (mostly using cannabis from illegal sources) found that 52% reported adverse side effects of MC use (mostly mild to moderate) including changes to appetite (29%), drowsiness (12%), irritated eyes (11%), poor memory (10%) and fatigue (9%), while 10% self‐reported dependency [52]. An Israeli study found that of 4891 patients that had initiated prescribed MC treatment (mostly legal sourced), 34.2% reported experiencing a side‐effect at their 6‐month follow up including dizziness (8.2%), dry mouth (6.7%), greater appetite (4.7%), tiredness (4.4%) and intoxicating effect (4.3%) [53].

Parents in our study took active steps to prevent youth exposure to cannabis by consuming MC in the evening or at night and discussing with their children the medicinal purposes of their cannabis use. However, parental use and attitudes towards cannabis may influence future cannabis use among their children. A Slovenian study of children aged 14 to 21 years (*n* = 839) found that 42.5% had used cannabis if their mothers were more tolerant of cannabis use, compared to 21.4% whose mothers did not permit cannabis use. Adolescents were also more likely to use cannabis (81%) if their mothers had used it, compared to those whose mothers did not use it (24.3%) [54]. Studies have noted the higher health risks of frequent cannabis use, particularly high THC potency, among youth including cognitive impairment, addiction, poor academic performance, adverse impacts on neurodevelopment, and associations with mental health conditions such as depression and suicidality [55, 56, 57]. These studies do not distinguish between recreational and medical cannabis use but it is reasonable to assume health risks related to frequent, high potency use of cannabis are relevant for risks related to medical use. In NZ, two longitudinal studies found that while most recreational cannabis users experienced little to no harm from use, a subset of the population who started consuming cannabis from early to mid‐adolescence were at risk of developing adult psychosis, particularly when they had a genetic vulnerability [58].

Mothers' in‐depth consideration of discussing MC with children and anticipating these conversations was a novel finding of this research. Most prior studies [59, 60] have depicted parent–child conversations about cannabis to be about adverse impacts, developmental issues and monitoring youth behaviours. In contrast, most mothers in our study employed a destigmatising approach to cannabis use in these conversations [61]. They did this by classifying cannabis in the same category as their other medications and discussing the therapeutic benefits with their children, with a handful albeit cautious about having these conversations too early. This raises questions about how MC is being uniquely conceptualised by parents. Parents may actively be working to destigmatise MC, distancing it from cannabis' previous prohibited status and the associated negative societal perceptions of cannabis consumers. Participants had not shared their MC with their children, but suggested they were open to considering cannabis treatment as an option for them if they needed it in the future. By engaging children in conversations about MC, parents may be reflecting and contributing to shifting cultural attitudes that increasingly recognise cannabis as a medicine. Natural and cultural narratives that normalise cannabis use for medicinal purposes may also be adding to these perceptions, influenced by the global legalisation of MC, and may also be tied to the increasing number of parents who believe cannabis should be available to medically vulnerable children as a treatment [27, 62]. Other studies demonstrate that cannabis drug education in schools also encourages a harm reduction approach, including an understanding of the medicinal properties of cannabis by teachers [63, 64].

Our findings support research from other studies [65, 66] showing that the high costs of legal MC products are a significant access barrier for women. Prior to the NZMCS, a survey found Māori and low‐income earners were less likely to transition to the scheme due to the anticipated high price of products [52]. Furthermore, we have previously commented that legal MC products would not be subsidised by the Pharmaceutical Management Agency (NZ government body responsible for funding medicines), and therefore financial barriers to accessing products were likely to persist for patients [2]. Most participants in this study had to resort to illegal or grey access routes such as the unregulated illegal market, social supply networks or growing MC themselves. This engendered feelings of worry, risk and frustration with the health system, particularly for mothers who were single and on low incomes. Mothers in our study excluded MC from their budgets for periods of time to prioritise the needs of their children or rationed their use. Maternal sacrifice to prioritise children's needs is well‐documented in public discourses as an integral part of the motherhood experience [67, 68]. Participants expressed the significant risks for mothers who are cannabis users, such as losing custody of their children [69, 70] and being labelled as a ‘bad mother’ by society [31]. Caucasian mothers who used MC felt their race intersected with their other social identities, that is, full‐time employment, married etc., to afford them privileged treatment by the criminal justice system, namely lower likelihood of criminal charges for illegal cannabis possession and a normalised view of cannabis consumption for this group [64]. This contrasted with rhetoric by a Māori mother in our study who felt cannabis use would be weaponised against Māori women to further marginalise and oppress them [33].

At the time of interviews, participants may have been unaware that the costs of legal MC products had been declining [71] and, for some products, were similar to the prices of cannabis in the illegal market. Participants may have also considered the additional costs of seeing their general practitioner to get a MC prescription or the cost of a consultation with a cannabis clinic as a financial barrier. Participants may have also not been aware that legal MC is often considered to be drier (98% dry) compared to illegal cannabis (typically 75%–80% dry), and though the higher moisture content in the latter makes it heavier, less of it is consumable cannabis material compared to prescribed products [72]. As well, participants that resort to the illegal market may incur health risks from consuming illegal produced cannabis due to unregulated CBD (cannabinol) and THC potencies, risk of contamination and toxins, and the presence of moulds and fungus [73, 74]. Limited perceived knowledge of legal MC product prices may be because most MC products in NZ are considered ‘unapproved’ medicines (except Sativex and Epidyolex) under NZ's Medicines Act 1981 and cannot be advertised [75] to minimise the harms of promoting medicines that demonstrate limited evidence of efficacy, safety and quality [76].

The women's stories reflected an internal conflict between wanting to empower themselves by using MC and discussing it with their children, but then consuming it after their children went to sleep so as not to expose them to it. Previous studies have observed that parents prefer to conceal their use of drugs from their children, in attempts to distance their child's construction of family life from their drug use [77, 78]. One study found parents did not want their children using cannabis despite their own medicinal use and therefore preferred to hide their consumption [19]. Among the mothers in our study, the stigma surrounding MC use [79, 80] was compounded by the double standards set for mothers by society. A few participants discussed how the internalisation of cannabis stigma stemming from its recent status as a prohibited drug and dated societal narratives in their upbringing made them question the legitimacy of their current use. While accessing a prescription mediated feelings of doubt and facilitated self‐confidence with consuming MC, stigma also prevented a few participants from discussing MC with their children, friends and family for fear others might discover it and judge them. Most participants, however, denounced cannabis stigma by asserting personal counternarratives of empowerment [22], which included discussing their MC use openly with their children and other mothers.

### 4.1 Limitations

This study set out to conduct in‐depth interviews with women about their MC use during the early years of NZ's new MC scheme. The limitation of the in‐depth qualitative approach is the small study sample size. This sample does not represent the views of all mothers from different ethnicities, younger age cohorts, and social backgrounds and identities using MC in Aotearoa NZ. Virtual interviews allowed interviews to be conducted at a time and date convenient for the participants, however, they may not cover detail that could have been attained from in‐person interviews.

## 5 CONCLUSION

This study has provided insights into MC use among mothers, highlighting perceived therapeutic benefits for managing the unique stressors of motherhood and health and wellbeing in general. The findings illustrate the global legalisation of MC as a possible catalyst for shifting attitudes towards cannabis use in parenting, and a trend of women exercising agency in their health using complementary alternative therapies. They also highlight the importance of developing guidelines that support discussions with healthcare providers about MC and policies that address barriers for mothers wanting to access legal MC products. This need is underscored by the unique risks mothers face when using illegal access routes for MC, such as discrimination in the legal system and losing custody of their children. As the global MC landscape evolves and women grow as a key demographic, future research should further investigate the nuances and complexities of mothers seeking and using MC and the impacts of MC use on parenting.

## AUTHOR CONTRIBUTIONS

Vinuli Withanarachchie designed the research, collected data, contributed to the analysis and writing. Associate Professor Marta Rychert acquired funding, contributed to the research design, analysis, writing and editing. Professor Chris Wilkins acquired funding, contributed to the research design, writing and editing.

## CONFLICT OF INTEREST STATEMENT

No competing interests.

## ETHICS STATEMENT

The Massey University Human Ethics Ohu Matatika 1 reviewed the study protocol and provided ethics approval (OM1 23/50). The study was performed in accordance with ethical standards laid down in the 1964 Declaration of Helsinki and its later amendments. Informed consent was gained from all participants before the interview.

## Data Availability

Research data are not shared.
